# Genome sequence analysis of multidrug-resistant *Mycobacterium tuberculosis* from Malaysia

**DOI:** 10.1038/s41597-020-0475-x

**Published:** 2020-05-05

**Authors:** Joon Liang Tan, Alfred Simbun, Kok-Gan Chan, Yun Fong Ngeow

**Affiliations:** 1 Faculty of Information Science and Technology, Multimedia University, Melaka, Malaysia; 2 Alfred Initiatives, 3 Elements SOHO, A-13-22 Taman Prima Tropica, 43300 Seri Kembangan, Selangor Malaysia; 3 Institute of Biological Sciences, Faculty of Science, University of Malaya, 50603 Kuala Lumpur, Malaysia; 4 International Genome Centre, Jiangsu University, Zhenjiang, China; 5 Department of Pre-Clinical Sciences, Faculty of Medicine and Health Sciences, Universiti Tunku Abdul Rahman, Bandar Sungai Long, Kajang, 43000 Selangor, DE Malaysia

**Keywords:** Computational biology and bioinformatics, Microbiology

## Abstract

*Mycobacterium tuberculosis* (MTB) is commonly used as a model to study pathogenicity and multiple drug resistance in bacteria. These MTB characteristics are highly dependent on the evolution and phylogeography of the bacterium. In this paper, we describe 15 new genomes of multidrug-resistant MTB (MDRTB) from Malaysia. The assessments and annotations on the genome assemblies suggest that strain differences are due to lineages and horizontal gene transfer during the course of evolution. The genomes show mutations listed in current drug resistance databases and global MTB collections. This genome data will augment existing information available for comparative genomic studies to understand MTB drug resistance mechanisms and evolution.

## Abstract (summary)

**Table:** 

Measurement(s)	DNA • genome • sequence_assembly • sequence feature annotation
Technology Type(s)	DNA sequencing • sequence assembly process • sequence annotation
Factor Type(s)	strain
Sample Characteristic - Organism	Mycobacterium tuberculosis
Sample Characteristic - Location	Malaysia

Machine-accessible metadata file describing the reported data: 10.6084/m9.figshare.12130008

## Background & Summary

Tuberculosis (TB) is still a public health challenge in many parts of the world. In Malaysia, an upper-middle income country in South-East Asia with a population of a little over 32 million, the TB incidence is still estimated as 92 per 100,000 population in 2019 (https://www.who.int/tb/country/data/profiles/en/), despite having active TB prevention programmes in place since the 1960s. Fortunately, the incidence of multidrug-resistant TB (MDRTB) has remained relatively low at about 1.5% of new TB cases and 3.1% of treated infections, and there have been only two reports of extensively drug-resistant TB (XDRTB) since 2015^1,2^. Nevertheless, the increasing detection of drug-resistant TB (DRTB) has raised concerns and prompted more vigorous surveillance and control strategies to prevent further escalation of the drug resistance problem. Specific control measures include the routine screening of foreign workers from high TB burden countries as official data showed that foreigners contributed to about 12–14% of TB in the country (http://www.moh.gov.my)^3^.

The GENEXPERT MTB/RIF testing of sputum samples is widely used in major hospitals in the country. Rapid molecular assays for the detection of resistance-associated mutations are also available for the testing of MTB isolates in TB laboratories. In addition, there is increasing awareness of the advantage of using whole genome sequencing to study drug resistance patterns and mechanisms. In this study, we analysed the genomes of 15 local isolates of MDRTB and compared them with drug-susceptible TB (DSTB) and MDRTB from other parts of the world. With these comparisons we hope to expand our understanding of the genetic determinants of drug resistance in MTB as well as the evolution of drug resistance in these bacteria.

## Methods

### MDRTB strains

The 15 MDRTB genomes used in this study were extracted from archived strains isolated from patients with pulmonary tuberculosis who were recruited for a study on TB molecular epidemiology approved by the University Malaya Medical Centre (UMMC) Medical Research Ethics Committee (MREC) (reference no. 975.28). The UMMC MREC does not require a separate approval for the use of archived bacterial isolates from clinical specimens.

The patients were referred to the tertiary care reference hospital from several states in Malaysia, from 2009–2012. The archived isolates were recovered with the BACTEC MGIT 960 liquid culture system (Becton Dickinson), re-identified and tested for rifampicin and isoniazid resistances using the Hain Genotype MTBDRplus Line Probe Assay (Hain Lifescience GmbH, Germany) according to the manufacturer’s instructions.

### Generation of draft genomes

For whole-genome sequencing, subcultures on Lowenstein-Jensen slants were heat inactivated at 80 °C for 2 h, and cooled down to room temperature before DNA extraction using the phenol/Chloroform/Isoamylalcohol (PCI) method^4^. DNA of adequate quality and quantity was used for sequencing using the MiSeq platform.

The sequencing reads were quality controlled using Trimmomatic^5^. The reads with quality lower than Q20 were removed prior to assembly. The clean reads were assembled in Vague assembler^6^. Each assembly was conducted using multiple k-mer sizes. Only the assemblies with the highest N50 and genome size of approximately 4 × 10^6^ bases were selected. The assemblies were then polished using error-corrected Illumina reads with bwa (parameter: *index*; *mem*), SAMTOOLS (parameter: *view –bS*; *sort*) and PILON 1.17(parameter: *–fix all*; –*genome H37Rv*)^7–9^.

### Genome annotation and analysis

The genomes were annotated in Prokka-Roary-Piggy pipelines with recommended guidelines^10–12^. In short, structural annotation for ORF prediction was conducted in Prokka (parameter: *–gffver 3*; *–kingdom bacteria*; *–evalue 1e-06*) and the output in the GFF3 format was subjected to Roary. In Roary, the core and accessory genomes of the Malaysian MDRTB were identified (parameter: *-I 95*; *-e*; *-n*). The annotation output from Prokka, together with the pan-genome analysis in Roary was used for intergenic regions prediction in Piggy (parameter: *-n 90*; *-l 90*; *-m g*). The lineages of the strains were predicted using the polished scaffold in TB Profiler web server^13^, based on the SNP barcode derived from 1,601 genomes^14^.

Protein sequences yielded from the genome annotation were submitted to the Comprehensive Antibiotic Resistance Database (CARD 2017)^15^. In addition, the Malaysian MDRTB genomes were compared with the collection of MDRTB genomes from multiple countries as deposited in the Tuberculosis Antibiotic Resistance Catalog Project (TB-ARC) of the Broad Institute (accessed on February 2019). The project is now hosted by NCBI and searchable with the keyword “TB-ARC”. The data files for more than 1000 MDRTB strains were downloaded for comparative genomics analysis using the variant calling methods described by Manson and colleagues^16^.

## Data Records

The draft genomes of the 15 Malaysian strains were captured with sequencing reads ranging from approximately 4.9 × 10^6^ to 7.6 × 10^6^ sequences, with a mean sequencing depth of 135.7 × per genome. The genome sizes ranged from approximately 4.2Mbps to 4.3Mbps (Table 1). The genome sequences have been made available in GenBank with the accession numbers VASA00000000 to VASF00000000 and VARR00000000 to VARZ00000000^17^, and SRA identifier SRP223277^18^.

**Table 1:** Genome Overview of the 15 MDRTB.

Strain	Genome Size (bp)	N50 (bp)	#Contig	#CDS	Lineage
103	4,278,889	68,530	170	4401	2.2.1
105	4,281,806	60,231	182	4402	2.2.1
106	4,282,240	71,491	165	4453	2.2.1
107	4,279,413	72,657	167	4442	2.2.1
108	4,275,219	72,767	163	4395	2.1
109	4,276,209	70,168	165	4408	2.1
110	4,261,340	42,986	212	4379	2.1
111	4,250,037	64,525	175	4340	2.1
112	4,,307,983	73,646	165	4414	1.1.3
113	4,269,918	79,261	153	4446	4.3.4.1
114	4,274,285	72,713	155	4390	2.2.1
115	4,257,368	18,772	439	4564	2.2.1
116	4,300,269	65,873	165	4401	1.1.3
117	4,322,642	79,033	149	4454	1.2.2
119	4,214,818	27,899	362	4456	2.2.1

## Technical Validation

### Whole genome data

The reliability of the genomes yielded from this study was assessed based on multiple aspects. The sequences were compared to the established reference genome of *M. tuberculosis* H37Rv^19^. With a number of contigs ranging from 149 to 439, the sequencing covered an average of 99.26% of the H37Rv genome (Table 1). Pan-genome analysis also showed no significant differences in the distribution of coding sequences among the strains. A total of 3484 (about 97%) core gene families in the 15 MDRTB strains^17,20^ were observed in *M. tuberculosis* H37Rv. The remaining 100 core gene families shared among the 15 MDRTB strains but not with H37Rv, were mostly hypothetical proteins and PE protein families. The high similarity in genomic regions and contents supports the reliability of the yielded genomes.

The presence of accessory genomes was evaluated to understand the factors potentially contributing to the differences among the assemblies and *M. tuberculosis* H37Rv. Among the accessory proteins, eight CRISPR proteins were found in strains 112, 113, 116 and 117, seven in strains 108, 109 and 110, six in strain 107 and five in the remaining seven strains. The number of intergenic regions predicted in each of the 15 strains ranged from 2172 to 2288, of which, 1365 were shared by all 15 strains, 1453 shared by at least two strains and 974 were strain-specific. CRISPR and intergenic regions have been reported to be the sequences influenced by horizontal gene transfer^12^. These features appear to be the main contributory factors to the dissimilarities among the strains and *M. tuberculosis* H37Rv.

### Region of difference

An evaluation of the region of difference (RD)^21^ revealed diverse evolutionary histories among the 15 strains: seven were predicted to be of East Asian Lineage 2.2.1 by the deletions of RD105, RD181 and RD207; four showed the RD105 deletion that suggested East Asian Lineage 2.1.; two had the RD239 deletion associated with Indo-Oceanic Lineage 1.1.3; one strain was of Lineage 1.2.2, while one strain with RD174 and a 7 bp deletion of the pks15/1 was assigned to the Euro-American Lineage 4.3.4.1. All the strains showed intralineages SNP of >12, suggesting no epidemiological link among the strains^22^.

### Drug resistance

We sought to assess the genetic factors contributing to the drug resistance properties among the strains. On screening against curated collections of drug resistance genes in CARD, the genes found in all strains were the transcription regulator rbpA (except strain 119), the efflux gene efpA associated with the expression of rifampicin resistance, the response regulator mtrA that modulates drug susceptibility, the gyrA and gyrase inhibitor mfpA involved in fluoroquinolone resistance and the AAC(2′) linked with aminoglycoside resistance. All but one strain had the erythromycin methyltransferase erm(37) that confers resistance to streptogramin, lincosamide and macrolides (Online-only Table 1).

Additionally, shared variants associated with drug resistance were found. All strains had a C117D substitution in the murA gene known to contribute to fosfomycin resistance, and all had a S95T substitution in gyrA that is associated with fluoroquinolone resistance, with one strain having an additional S91P substitution. Twelve and three strains showed four (D516G, H526T, L511R, S450L) and three mutations (D516G, L511R, H526T) respectively, in the rpoB gene involved with rifampicin resistance. Other substitutions observed included a consistent A2274G mutation in the 23S rRNA that had been reported to confer clarithromycin resistance, and mutations in the embA, embB, embC, embR, katG, pncA, thyA and rpsL genes that are associated with phenotypic resistance in various anti-TB drugs (Online-only Table 1).

Genome-wide screening of coding regions with PhyResSE^23^ showed results that are congruent with those observed in the CARD analysis. In addition, variants upstream to known resistance conferring mutations were also identified. Intergenic mutations known to be associated with drug resistance were found in six strains. These comprised isoniazid resistance-associated mutations at the upstream of Rv1483 in strains 106, 109, 110, 113 and 115, and a single kanamycin resistance-associated mutation upstream to Rv2416c in strain 108.

Compared to H37Rv, the number of single nucleotide polymorphisms in the 15 Malaysian MDRTB strains ranged from 820 to 2230. Six nonsynonymous mutations and upstream variants located in known drug resistance genes were not matched with SNPs in the PhyResSE database. Global comparisons with TB-ARC showed all six variants could be found in at least one MDRTB from other countries. The two most commonly shared variants between the 15 Malaysian MDRTB and the global MDRTB are the variants G7362C and G9304A in the gyrA gene known to be responsible for fluoroquinolone resistance. No Malaysian-specific polymorphisms were observed on comparison with global strains in the TB-ARC.

Our genome-wide screening did not detect any evidence of XDRTB as defined by resistance to first-line anti-TB drugs plus any fluoroquinolone and at least one of three injectable second-line drugs (amikacin, kanamycin, or capreomycin). While all strains harboured the fluoroquinolone resistance (mfpA, gyrA) genes, none were found to have the genes for capreomycin (tlyA, Ins49GC), amikacin (rrs) and kanamycin (rrs; eis, whiB7, gidB). The AAC(2′) aminoglycoside acetyltransferase gene found in all strains has been reported to be universally present in many mycobacterial species^24^. It is unclear whether its presence together with fluoroquinolone resistance genes predisposed to the emergence of XDRTB in Malaysia, two years after the completion of this study.

## Usage Notes

Surrounded by high TB burden regions in SE Asia, Malaysia has to be vigilant against further dissemination of TB and MDRTB in the country. More in-depth analyses of genome sequence information will provide a better understanding of MDRTB genetics and transmission dynamics which may ultimately lead to more effective strategies for the clinical and public health management of TB.

## Online-only Table

**Online-only Table 1:** Results of phenotypic drug susceptibility tests and genotypic analysis of 15 MDRTB isolates from Malaysia.

Sample ID/type	Year isolated	1^st^ DST RISE	2^nd^ DST FACE	Drug Resistance Genes detected		
				Hain LifeSciences line probe assay		Best Hit ARO
				1^st^ line *rpoB, katG, inhA*	2^nd^ line *gyrA, rrs, embB*	
103/spt	2009	RRRS	SSSS	*rpoB, inhA*	Nil	*AAC(2*′*)-Ic*, *efpA*, *mfpA*, *mtrA*, 23S rRNA (clarithromycin), *gyrA*, *murA*, *katG*, *rpoB*, *erm(37)*, *rbpA*, *embB*, *rpsL*
105/spt	2009	RRRS	SSSS	ND	Nil	*AAC(2*′*)-Ic*, *efpA*, *mfpA*, *mtrA*, 23S rRNA (clarithromycin), *gyrA*, *murA*, *katG*, *rpoB*, *erm(37)*, *rbpA*, *embB*
106/spt	2009	RRRS	SSSS	*rpoB, katG, inhA*	Nil	*AAC(2*′*)-Ic*, *efpA*, *mfpA*, *mtrA*, 23S rRNA (clarithromycin), *gyrA*, *murA*, *katG*, *rpoB*, *erm(37)*, *rbpA*
107/spt	2009	RRSS	SSSS	*rpoB, inhA*	Nil	*AAC(2*′*)*, *efpA*, *mfpA*, *mtrA*, 23S rRNA (clarithromycin), *gyrA*, *murA*, *katG*, *rpoB*, *erm(37)*, *rbpA*, *embB*
108/spt	2010	RRRS	SSSS	*rpoB, katG*	Nil	*AAC(2*′*)*, *efpA*, *mfpA*, *mtrA*, 23S rRNA (clarithromycin), *gyrA*, *murA*, *katG*, *rpoB*, *erm(37)*, *rbpA*, *embB*
109/TS	2010	RRRS	SSSS	*rpoB, katG, inhA*	*embB*	*AAC(2*′*)*, *efpA*, *mfpA*, *mtrA*, 23S rRNA (clarithromycin), *gyrA*, *murA*, *katG*, *rpoB*, *erm(37)*, *rbpA*
110/CSF	2010	RRRS	SSSS	*rpoB, katG, inhA*	Nil	*AAC(2*′*)*, *efpA*, *mfpA*, *mtrA*, 23S rRNA (clarithromycin), *gyrA*, *murA*, *katG*, *rpoB*, *erm(37)*, *rbpA*, *pncA*
111/spt	2010	RRRS	SSSS	*rpoB, katG*	Nil	*AAC(2*′*)*, *efpA*, *mfpA*, *mtrA*, 23S rRNA (clarithromycin), *gyrA*, *murA*, *katG*, *rpoB*, *erm(37)*, *rbpA*
112/spt	2010	RRRS	SSSS	*rpoB, inhA*	Nil	*AAC(2*′*)*, *efpA*, *mfpA*, *mtrA*, 23S rRNA (clarithromycin), *gyrA*, *murA*, *katG*, *rpoB*, *erm(37)*, *rbpA*, *embA/B/C/R*, *rpsL*
113/spt	2010	RRRS	SSSR	*rpoB, inhA*	*embB*	*AAC(2*′*)*, *efpA*, *mfpA*, *mtrA*, 23S rRNA (clarithromycin), *gyrA*, *murA*, *katG*, *rpoB*, *erm(37)*, *rbpA*, *thyA*
114/spt	2011	RRRS	RSSS	*rpoB*	*gyrA, rrs, embB*	*AAC(2*′*)*, *efpA*, *mfpA*, *mtrA*, 23S rRNA (clarithromycin), *gyrA*, *murA*, *katG*, *rpoB*, *erm(37)*, *rbpA*, *embB*
115/spt	2011	RRSR	SSSS	*rpoB, katG*	*embB*	*AAC(2*′*)*, *efpA*, *mfpA*, *mtrA*, 23S rRNA (clarithromycin), *gyrA*, *murA*, *katG*, *rpoB*, *erm(37)*, *rbpA*
116/spt	2011	RRSR	SSSS	*rpoB, katG*	Nil	*AAC(2*′*)*, *efpA*, *mfpA*, *mtrA*, 23S rRNA (clarithromycin), *gyrA*, *murA*, *katG*, *rpoB*, *erm(37)*, *rbpA*, *embA/B/C/R*, *pncA*, *rpsL*
117/gl	2012	RRRR	SSSR	*rpoB, katG*	*gyrA*	*AAC(2*′*)*, *efpA*, *mfpA*, *mtrA*, 23S rRNA (clarithromycin), *gyrA*, *murA*, *katG*, *rpoB*, *erm(37)*, *rbpA*, *embA/B/C/R*
119/spt	2011	RRRS	SSSS	*rpoB*	Nil	*AAC(2*′*)*, *efpA*, *mfpA*, *mtrA*, 23S rRNA (clarithromycin), *gyrA*, *murA*, *katG*, *rpoB*

Spt, sputum.

TS, tracheal secretion

CSF, cerebrospinal fluid.

Gl, gastric lavage.

R, resistant.

S, susceptible.

1^st^ 1ine DST: drug susceptibility testing using the Bactec MGIT 960 System for first-line drugs.

RISE: rifampicin, isoniazid, streptomycin, ethambutol.

ARO: Antibiotic Resistance Ontology.

1^st^ line drug mutations using GenoType MTBDR*plus* (Hain Lifescience GmbH, Germany) line probe assay targeting the *rpoB (*rifampicin*), katG (*isoniazid*)* and *inhA* (isoniazid) genes.

2^nd^ DST: drug susceptibility testing using the Bactec MGIT 960 System for second-line drugs.

FACE: fluoroquinolone (ofloxacin), amikcin, capreomycin, ethambutol.

2^nd^ line drug mutations using GenoType MTBDR*sl* (Hain Lifescience Gmbh, Germany) line probe assay targeting the *gyrA (*fluoroquinolones*), rrs (*amikacin/capreomycin*)*, and *embB* (ethambutol)genes.
